# Supplementary material for: A distinct transition from cell growth to physiological homeostasis in the tendon
Source: eLife. 2019 Sep 19;8:e48689. doi: 10.7554/eLife.48689 (PMC6791717; doi:10.7554/eLife.48689)
Supplement: Supplementary file 1. [file elife-48689-supp1.pdf]

Supplementary Table 1: RT-qPCR Statistics

Grinstein et al.

April 23, 2019

Supp. Table 1A: Ki67 ANOVA

	**df**	**sum sq**	**mean sq**	**F statistic**	**p value**
Timepoint	5	71.751570	14.3503140	16.01767	**2e-04**
Residuals	10	8.959051	0.8959051	NA	NA

Supp. Table 1B: Ki67 Tukey's HSD

**comparison**	**estimate**	**conf. low**	**conf. high**	**adj. p value**
P14-P0	0.6183	-2.0660	3.3026	0.9611
P21-P0	3.6772	0.9929	6.3615	**0.0073**
P28-P0	2.1664	-0.8347	5.1675	0.209
P35-P0	5.9006	2.8994	8.9017	**5e-04**
P7-P0	-0.3139	-2.9982	2.3704	0.9981
P21-P14	3.0589	0.3746	5.7432	**0.024**
P28-P14	1.5481	-1.4531	4.5492	0.5103
P35-P14	5.2822	2.2811	8.2834	**0.0012**
P7-P14	-0.9322	-3.6165	1.7521	0.8247
P28-P21	-1.5108	-4.5120	1.4903	0.5336
P35-P21	2.2233	-0.7778	5.2245	0.1906
P7-P21	-3.9911	-6.6754	-1.3068	**0.0041**
P35-P28	3.7342	0.4466	7.0217	**0.0244**
P7-P28	-2.4803	-5.4814	0.5209	0.1239
P7-P35	-6.2144	-9.2156	-3.2133	**3e-04**

Supp. Table 1C: Scx ANOVA

	df	sum sq	mean sq	F statistic	p value
Timepoint	5	82.31887	16.463773	7.513398	**0.0021**
Residuals	12	26.29506	2.191255	NA	NA

Supp. Table 1D: Scx Tukey's HSD

comparison	estimate	conf. low	conf. high	adj. p value
P14-P0	-4.7794	-8.8392	-0.7197	**0.0183**
P21-P0	-0.6600	-4.7198	3.3998	0.9928
P28-P0	-1.4433	-5.5031	2.6164	0.8315
P35-P0	1.6778	-2.3820	5.7375	0.7332
P7-P0	-3.4111	-7.4709	0.6487	0.1206
P21-P14	4.1194	0.0597	8.1792	**0.046**
P28-P14	3.3361	-0.7237	7.3959	0.133
P35-P14	6.4572	2.3975	10.5170	**0.0019**
P7-P14	1.3683	-2.6914	5.4281	0.859
P28-P21	-0.7833	-4.8431	3.2764	0.9845
P35-P21	2.3378	-1.7220	6.3975	0.4285
P7-P21	-2.7511	-6.8109	1.3087	0.2744
P35-P28	3.1211	-0.9387	7.1809	0.1753
P7-P28	-1.9678	-6.0275	2.0920	0.5973
P7-P35	-5.0889	-9.1487	-1.0291	**0.0119**

Supp. Table 1E: Mkx ANOVA

	**df**	**sum sq**	**mean sq**	**F statistic**	**p value**
Timepoint	5	40.505138	8.1010277	9.78096	**7e-04**
Residuals	12	9.938935	0.8282446	NA	NA

Supp. Table 1F: Mkx Tukey's HSD

**comparison**	**estimate**	**conf. low**	**conf. high**	**adj. p value**
P14-P0	0.3294	-2.1665	2.8254	0.9973
P21-P0	1.8628	-0.6332	4.3587	0.1965
P28-P0	1.9011	-0.5948	4.3970	0.1818
P35-P0	3.8550	1.3591	6.3509	**0.0024**
P7-P0	-0.6239	-3.1198	1.8720	0.9539
P21-P14	1.5333	-0.9626	4.0293	0.365
P28-P14	1.5717	-0.9243	4.0676	0.3414
P35-P14	3.5256	1.0296	6.0215	**0.0049**
P7-P14	-0.9533	-3.4493	1.5426	0.7886
P28-P21	0.0383	-2.4576	2.5343	1
P35-P21	1.9922	-0.5037	4.4882	0.1506
P7-P21	-2.4867	-4.9826	0.0093	0.0511
P35-P28	1.9539	-0.5420	4.4498	0.1631
P7-P28	-2.5250	-5.0209	-0.0291	**0.0468**
P7-P35	-4.4789	-6.9748	-1.9830	**7e-04**

Supp. Table 1G: Col1a2 ANOVA

	df	sum sq	mean sq	F statistic	p value
Timepoint	5	57.78858	11.557716	12.75345	**2e-04**
Residuals	12	10.87490	0.906242	NA	NA

Supp. Table 1H: Col1a2 Tukey's HSD

comparison	estimate	conf. low	conf. high	adj. p value
P14-P0	-0.7878	-3.3986	1.8230	0.9047
P21-P0	0.1978	-2.4130	2.8086	0.9998
P28-P0	0.0972	-2.5136	2.7080	1
P35-P0	4.0594	1.4486	6.6703	**0.0023**
P7-P0	-1.6511	-4.2619	0.9597	0.3373
P21-P14	0.9856	-1.6253	3.5964	0.7961
P28-P14	0.8850	-1.7258	3.4958	0.8563
P35-P14	4.8472	2.2364	7.4580	**5e-04**
P7-P14	-0.8633	-3.4741	1.7475	0.8679
P28-P21	-0.1006	-2.7114	2.5103	1
P35-P21	3.8617	1.2509	6.4725	**0.0034**
P7-P21	-1.8489	-4.4597	0.7619	0.2372
P35-P28	3.9622	1.3514	6.5730	**0.0028**
P7-P28	-1.7483	-4.3591	0.8625	0.2849
P7-P35	-5.7106	-8.3214	-3.0997	**1e-04**

Supp. Table 1I: Col3a1 ANOVA

	df	sum sq	mean sq	F statistic	p value
Timepoint	5	28.020784	5.6041568	15.16279	**1e-04**
Residuals	12	4.435193	0.3695994	NA	NA

Supp. Table 1J: Col3a1 Tukey's HSD

comparison	estimate	conf. low	conf. high	adj. p value
P14-P0	-0.1417	-1.8090	1.5257	0.9997
P21-P0	1.6956	0.0282	3.3629	**0.0455**
P28-P0	0.8817	-0.7857	2.5490	0.5133
P35-P0	2.6350	0.9677	4.3023	**0.002**
P7-P0	-1.1400	-2.8073	0.5273	0.2667
P21-P14	1.8372	0.1699	3.5045	**0.0281**
P28-P14	1.0233	-0.6440	2.6907	0.3659
P35-P14	2.7767	1.1093	4.4440	**0.0013**
P7-P14	-0.9983	-2.6657	0.6690	0.39
P28-P21	-0.8139	-2.4812	0.8534	0.5907
P35-P21	0.9394	-0.7279	2.6068	0.4502
P7-P21	-2.8356	-4.5029	-1.1682	**0.0011**
P35-P28	1.7533	0.0860	3.4207	**0.0374**
P7-P28	-2.0217	-3.6890	-0.3543	**0.015**
P7-P35	-3.7750	-5.4423	-2.1077	**1e-04**

Supp. Table 1K: Fmod ANOVA

	df	sum sq	mean sq	F statistic	p value
Timepoint	5	23.67170	4.734340	3.998559	**0.0229**
Residuals	12	14.20814	1.184012	NA	NA

Supp. Table 1L: Fmod Tukey's HSD

comparison	estimate	conf. low	conf. high	adj. p value
P14-P0	-2.8639	-5.8481	0.1203	0.0627
P21-P0	-2.3533	-5.3376	0.6309	0.1583
P28-P0	-2.4367	-5.4209	0.5476	0.1368
P35-P0	-0.5256	-3.5098	2.4587	0.9897
P7-P0	-2.8933	-5.8776	0.0909	0.0593
P21-P14	0.5106	-2.4737	3.4948	0.991
P28-P14	0.4272	-2.5570	3.4115	0.996
P35-P14	2.3383	-0.6459	5.3226	0.1625
P7-P14	-0.0294	-3.0137	2.9548	1
P28-P21	-0.0833	-3.0676	2.9009	1
P35-P21	1.8278	-1.1565	4.8120	0.368
P7-P21	-0.5400	-3.5242	2.4442	0.9884
P35-P28	1.9111	-1.0731	4.8953	0.3256
P7-P28	-0.4567	-3.4409	2.5276	0.9946
P7-P35	-2.3678	-5.3520	0.6165	0.1544
