# Supplementary material for: A distinct transition from cell growth to physiological homeostasis in the tendon
Source: eLife. 2019 Sep 19;8:e48689. doi: 10.7554/eLife.48689 (PMC6791717; doi:10.7554/eLife.48689)
Supplement: Supplementary file 2. [file elife-48689-supp2.docx]

**Supplementary File 2:** **List of RT-qPCR primers used in this study.**

| Target Gene | Forward Sequence (5’–3’) | Reverse Sequence (5’–3’) | Primer Source |
| --- | --- | --- | --- |
| *Col1a2* | CCAGCGAAGAACTCATACAGC | GGACACCCCTTCTACGTTGT | (Mendias et al., 2008) |
| *Col3a1* | TGACTGTCCCACGTAAGCAC | GAGGGCCATAGCTGAACTGA | Self-designed using PrimerBlast (Ye et al., 2012) |
| *Fmod* | CATGGCAACCAGATTACC | AGATATAAGGCCGTGAGG | (Alberton et al., 2015) |
| *Gapdh* | TGTTCCTACCCCCAATGTGT | GGTCCTCAGTGTAGCCCAAG | (Szymaniak et al., 2015) |
| *Mki67* | AGCAAGCCAACAGAATTTCCAG | TATCTTGACCTTCCCCATCAGG | Self-designed using PrimerBlast |
| *Mkx* | AGTGGCTTTACAAGCACCGT | TTTGACACCTGCACTAGCGT | Self-designed using PrimerBlast |
| *Scx* | AAGTTGAGCAAAGACCGTGAC | AGTGGCATCCACCTTCACTA | Self-designed using PrimerBlast |
